# Anticancer Effects of 15d-Prostaglandin-J_2_ in Wild-Type and Doxorubicin-Resistant Ovarian Cancer Cells: Novel Actions on SIRT1 and HDAC

**DOI:** 10.1371/journal.pone.0025192

**Published:** 2011-09-21

**Authors:** Edwin de Jong, Peter Winkel, Klaas Poelstra, Jai Prakash

**Affiliations:** 1 Department of Pharmacokinetics, Toxicology and Targeting, Groningen Research Institute for Pharmacy, University of Groningen, Groningen, The Netherlands; 2 BiOrion Technologies BV, MediTech Center UMCG, L.J. Zielstraweg 1, Groningen, The Netherlands; Ludwig-Maximilians University, Germany

## Abstract

15-deoxy-delta-12,14-prostaglandin-J_2_ (15d-PGJ_2_), an arachidonic metabolite and a natural PPARγ agonist, is known to induce apoptosis in tumor cells. In this study, we investigated new therapeutic potentials of 15d-PGJ_2_ by determining its anticancer effects in wild-type and doxorubicin-resistant ovarian carcinoma cells. Despite high expression of resistance-inducing genes like MDR1, Bcl2 and Bcl-xl, 15d-PGJ_2_ strongly induced apoptosis in doxorubicin-resistant (A2780/AD) cells similar to the wild-type (A2780). This was found to be related to caspase-3/7- and NF-κB pathways but not to its PPARγ agonistic activity. 15d-PGJ_2_ also was able to reduce the doxorubicin resistance of A2780/AD cells at low doses as confirmed by the inhibition of gene expression of MDR1 (p-glycoprotein) and SIRT1 (a drug senescence gene). We also investigated effects of 15d-PGJ_2_ on cell migration and transformation using a wound-healing assay and morphological analyses, respectively. We found that 15d-PGJ_2_ inhibited migration most likely due to NF-κB inhibition and induced transformation of the round-shape A2780/AD cells into elongated epithelial cells due to HDAC1 inhibition. Using a 15d-PGJ_2_ analog, we found the mechanism of action of these new activities of 15d-PGJ_2_ on SIRT1 and HDAC1 gene expressions and enzyme activities. In conclusion, the present study demonstrates that 15d-PGJ_2_ has a high therapeutic potential to kill drug-resistant tumor cells and, the newly described inhibitory effects of this cyclo-oxygenase product on SIRT1 and HDAC will provide new opportunities for cancer therapeutics.

## Introduction

Prostaglandins (PGs) are a family of biologically active endogenous metabolites of arachidonic acid. They control a vast variety of physiological functions such as regulation of smooth muscle tone, inflammation, cellular growth and differentiation [1]. 15-deoxy-Δ^12,14^-prostaglandin J_2_ (15d-PGJ_2_) is a dehydration derivative of PGD_2_, which is also known as a natural agonist of the nuclear receptor peroxisome proliferator−activated receptor gamma (PPARγ). 15d-PGJ_2_ has been reported to display multiple pharmacological activities (anti-inflammatory, anti-fibrotic and apoptotic activities) either through PPARγ−dependent pathways or PPARγ–independent pathways such as Nuclear Factor-kappaB (NF-κB)−, Keap-Nrf2−, STAT1, and p53−dependent pathways [2], [3]. In our recent study, we have shown that 15d-PGJ_2_ was able to inhibit tumor progression significantly *in vivo* in tumor-bearing mice. Its effectivity was found to be controlled by its strong but reversible serum albumin binding and by the subsequent penetration of albumin into the tumors which was dependent on the tumor vasculature [4]. Also other groups have reported the anti-tumor activity of 15d-PGJ_2_ in vivo in different tumor models [5], [6]. These results suggest that a potential use of 15d-PGJ_2_ for therapeutic purposes as an anticancer agent can be envisioned.

We have shown that 15d-PGJ_2_ induces apoptosis in different cancer cells through PPARγ-independent NF-κB and caspase-dependent pathways [4], as also shown by other studies [7], [8]. Knowing the involvement of NF-κB pathway in the regulation of multidrug resistance (MDR1) and anti-apoptotic genes (Bcl-2 and Bcl-xl) [9], we now aimed to investigate whether 15d-PGJ_2_ is capable of inducing apoptosis in doxorubicin-resistant cancer cells compared to the wild-type. We further examined whether the effects induced by 15d-PGJ_2_ were mediated through PPARγ-dependent and/or NF-κB-dependent pathways. Chu and co-workers have demonstrated that Silent Information Regulator Type 1 (SIRT1), a class III histone deacetylase (HDAC), is over-expressed in various chemoresistant tumors of cancer patients and inhibition of SIRT1 gene expression leads to decrease in MDR1 expression and increase in drug sensitivity [10]. We therefore compared the SIRT1 expression in human wild-type and doxorubicin resistant ovarian cancer cells and examined the effects of 15d-PGJ_2_ on this SIRT1 gene expression. During these experiments, we noticed that 15d-PGJ_2_−treated doxorubicin-resistant cells transformed from round-shaped cells to an elongated type. We further investigated this phenotypic change and found that 15d-PGJ_2_ induced these effects by inhibiting class-I HDAC enzymes. Many pharmacological activities of 15d-PGJ_2_ e.g. inhibition of PPARγ, NF-κB, p53 and Nrf-keap pathways are induced by making a stable complex with free cysteine in these proteins through its one of the electrophilic carbon atoms [11]. In order to determine whether inhibitory effects of 15d-PGJ_2_ on SIRT1 and HDACs were also related to the latter mechanism, we performed several *in vitro* experiments using an analog of 15d-PGJ_2_. Our results show many new activities of this endogenous arachidonic acid metabolite on cancer cells and illuminate the mechanism of action of this cyclo-oxygenase product.

## Methods

### Cell experiments

Wild-type (A2780) and doxorubicin-resistant (A2780/AD) human ovarian carcinoma cell lines were obtained from University Medical Centre Groningen, The Netherlands. A2780 and A2780/AD (doxorubicin-resistant) cell lines were maintained on Dulbecco's modified Eagle's medium (DMEM, BioWhittaker, Verviers, Belgium) supplemented with 10% fetal calf serum (FCS) and antibiotics (penicillin, 50 units/ml plus streptomycin, 50 ng/ml) at 37°C in a humidified incubator containing 5% CO_2_. A2780/AD cells were cultured in the presence of doxorubicin (2 µM). Two weeks before the experiments a separate flask of doxorubicin-resistant cells was maintained without doxorubicin. These cells were used for further experiments to avoid the influence of doxorubicin on our experiments. Since 15d-PGJ_2_ in vitro looses its activity in the presence of FCS, all experiments were performed in FCS-free medium.

#### Cell viability studies

Cells were seeded into the 96-well plate as 1×10^4^ cells/well in 200 µl medium with 10% FCS. After 48 h, cells were washed with serum-free medium and then incubated with different concentrations of 15d-PGJ_2_ in serum-free medium for 48 h. In case of treatment with the irreversible PPARγ antagonist GW9662 (2-chloro-5-nitrobenzanilide, sigma), cells were pre-incubated with GW9662 (10 µM) for 3 h and then incubated with a mixture of 15d-PGJ_2_ (Cayman chemicals, Ann Arbor, MI) and GW9662 for 48 h. Viability of the cells was determined using Alamar Blue dye (Serotec, Oxford, UK) which measures the number of cells on the basis of mitochondrial activity. After 48 h of the incubation, medium containing the Alamar blue dye (diluted 1∶10) was added to the cells and incubated for another 4 h. Thereafter the metabolized dye (fluorescent) was detected with a fluorimeter at Excitation 560 nm and Emission 590 nm.

#### Caspase 3/7 enzyme assays

Caspase−3 and −7 enzymes activity was determined using Caspase 3/7 Glo assay kit (Promega, Medison, WI). 1×10^4^ cells were seeded in 96-well plate in 200 µl culturing medium. After 48 h, cells were washed with serum-free medium and incubated with different concentrations of 15d-PGJ_2_ in 100 µl medium for 5.5 h. Subsequently, 100 µl of the Caspase 3/7-reconstituted reagent was added to the cells and incubated for 30 min in the incubator. The luminescence was determined by a luminometer (Lumicount, Packard, Meriden, CT).

#### Annexin V staining

Annexin V staining was performed on the cells to determine the induction of apoptosis after treatment with 15d-PGJ2. 1×10^4^ cells were seeded in an 8-well plate (Lab-tek, Nunc, Roskilde, Denmark) in 400 µl culturing medium. After 72 h, cells were washed with serum-free medium and incubated with different concentrations of 15d-PGJ_2_ in 200 µl medium for 6 h. Subsequently, cells were incubated with 100 µl of the annexin V-FITC (Roche, Mannheim, Germany) for 15 min at room temperature, washed and fixed with 4% paraformaldehyde. Then, cells were mounted with DAPI-containing mounting solution and staining was examined under a fluorescent microscope.

#### Gene expression study

1 × 10^5^ cells per well were seeded in 12-well plate and cultured for 48 h and then incubated with different compounds for 48 h. Cells were lysed using a lysis buffer and RNA was isolated using Absolutely RNA microprep kit (Stratagene, La Jolla, CA) according to manufacturer's instructions. The RNA concentrations were quantitated by a UV spectrophotometer (NanoDrop Technologies, Wilmington, DE). Then cDNA was synthesized from equal amounts of RNA using Superscript III first strand synthesis kit (Invitrogen, Carlsbad, CA). The primers for human species were obtained from Sigma-Genosys (Haverhill, UK). The primer sequences are enlisted in Table 1. Gene expression levels for different genes were measured by quantitative real-time RT-PCR (Applied Biosystems, Foster City, CA) using SYBR Green as a fluorescent probe (Applied Biosystems). Finally, the threshold cycle numbers (Ct) were used to calculate the gene expression for each target by normalizing to the house-keeping gene GAPDH.

**Table 1:** Primer sequences for Real-time RT-PCR.

Gene symbols	Forward sequence	Reverse sequence
GAPDH	ACCCAGAAGACTGTGGATGG	TCTAGACGGCAGGTCAGGTC
Bcl-2	GTCTGGGAATCGATCTGGAA	AATGCATAAGGCAACGATCC
Bcl-xl	TCTGGTCCCTTGCAGCTAGT	TCCTTTCTGGGGAAGAGGTT
MDR1	AATGCGCCAAGACTAGGAAG	ACCGGAGGATGTTGAACAAG
Sirt1	GCAGATTAGTAGGCGGCTTG	TCTGGCATGTCCCACTATCA
HDAC1	GGAAATCTATCGCCCTCACA	AACAGGCCATCGAATACTGG
HDAC2	AGACTGCAGTTGCCCTTGAT	TGCGCAAATTTTCAAACAAA
HDAC3	TGGCTTCTGCTATGTCAACG	CCCGGTCAGTGAGGTAGAAA
Snail	ACCCCACATCCTTCTCACTG	TACAAAAACCCACGCAGACA
E-cadherin	TGCCCAGAAAATGAAAAAGG	GTGTATGTGGCAATGCGTTC

#### Transfection and Luciferase assay for NF-κB activity

NF-κB activity was determined using a Luciferase reporter based assay as described earlier [4]. In brief, pNF-κB-Luc (Clontech, Mountain View, CA) containing a specific binding sequence for NF-κB and an empty Luciferase plasmid, pTAL-Luc (control) were used for transfection studies. 1×104 cells per well were seeded in white 96-well plates and the transfection of the plasmids was carried out using FuGENE 6 transfection Reagent (Roche) after 24 h. Cells were treated with a complex of 0.17 µg DNA/0.5 µl FuGENE 6 in 100 µl normal media with 10% FCS for 24 h. Subsequently, cells were washed with serum-free media and incubated with 15d-PGJ_2_, BAY 11–7082 (Sigma), ciglitazone with or without TNF-α (Peprotech, Rocky Hill, NJ) for 4 h. Thereafter, cells were washed with PBS and lysed with 20 µl cell lysis buffer, and supplemented with 100 µl Luciferase substrate (Promega). Luciferase activity was measured by a luminometer (Lumicount, Packard). The luminescence unit values of pNF-κB-Luc were neutralized by subtracting the pTAL-Luc values.

#### Wound healing assay

1 × 10^5^ cells per well were seeded in 12-well plate and cultured for 48 h in culture medium. Thereafter, a scratch (wound) was introduced in the confluent cell layer using a yellow tip placed in a scaffold, allowing standardization of the scratch. Cells were washed three times with medium to remove detached cells. Cells were then incubated with different compounds for 48 h and pictures of a defined wound spot were made with computer-aided phase contrast microscope (Olympus) at t = 0, 24 and 48 h. The area of the wound in the microscopic pictures was measured using Image J software (National Institutes of Health, MD) at different time points. The percentage wound healing after 24 h or 48 h was calculated in relative to the total wound area at t = 0 h of the same wound spot.

#### SIRT1 and HDAC1 enzyme activity assays

SIRT1 and HDAC1 enzyme inhibition assays were performed to determine the effect of 15d-PGJ2 on their activities using the commercial kits from Cayman Chemicals (Ann Arbor, MI). The SIRT1 and HDAC1 enzyme assays were performed in microplates according to the instructions of the manufacturer. First, assay buffer, SIRT1 or HDAC1 enzyme and solvent (DMF) or different concentrations of treatments (15d-PGJ2 or CAY-10410 dissolved in DMF) were mixed. CAY-10410 (9,10-dihydro-15-deoxy-Δ^12,14^-Prostaglandin J_2_), an analog of 15d-PGJ_2_, was purchased from Cayman chemicals. Then, the substrate comprised of the p53 sequence Arg-His-Lys-Lys(e-acetyl)-AMC peptide and co-substrate NAD+ for SIRT1 activity was added to the enzyme mixture and incubated at room temperature for 45 min. To measure HDAC1 activity, an acetylated lysine substrate was added to the enzyme mixture and incubated for 30 min at room temperature. Thereafter, the stop/developing solution, containing a mixture of a developer and nicotinamide (SIRT1 inhibitor) or Trichostatin A (HDAC1 inhibitor) were added to the microplate and incubated for 30 min and 15 min, respectively at room temperature. Deacetylated peptide reacts with the developer and releases a fluorophore. The fluorophores for both assays were analyzed using an excitation wavelength of 350 nm and an emission wavelength of 450 nm. 100% activity of the enzyme was calculated by incubating with DMF alone and the percentage inhibition of SIRT1 or HDAC1 activity was calculated relative to the corresponding solvent concentrations.

### Statistical Analyses

Data are presented as mean ± standard error mean (SEM) unless otherwise mentioned. The statistical analyses were performed using unpaired two-tailed student's t-test with *p*<0.05 as the minimal level of significance. Cell-viability data were fitted according to sigmoidal dose-response curve to calculate IC_50_ using GraphPad Prism 4 software (La Jolla, CA).

## Results

### 15d-PGJ_2_ induces apoptosis in both A2780 and A2780/AD cells PPARγ-independently

**Figure 1:**
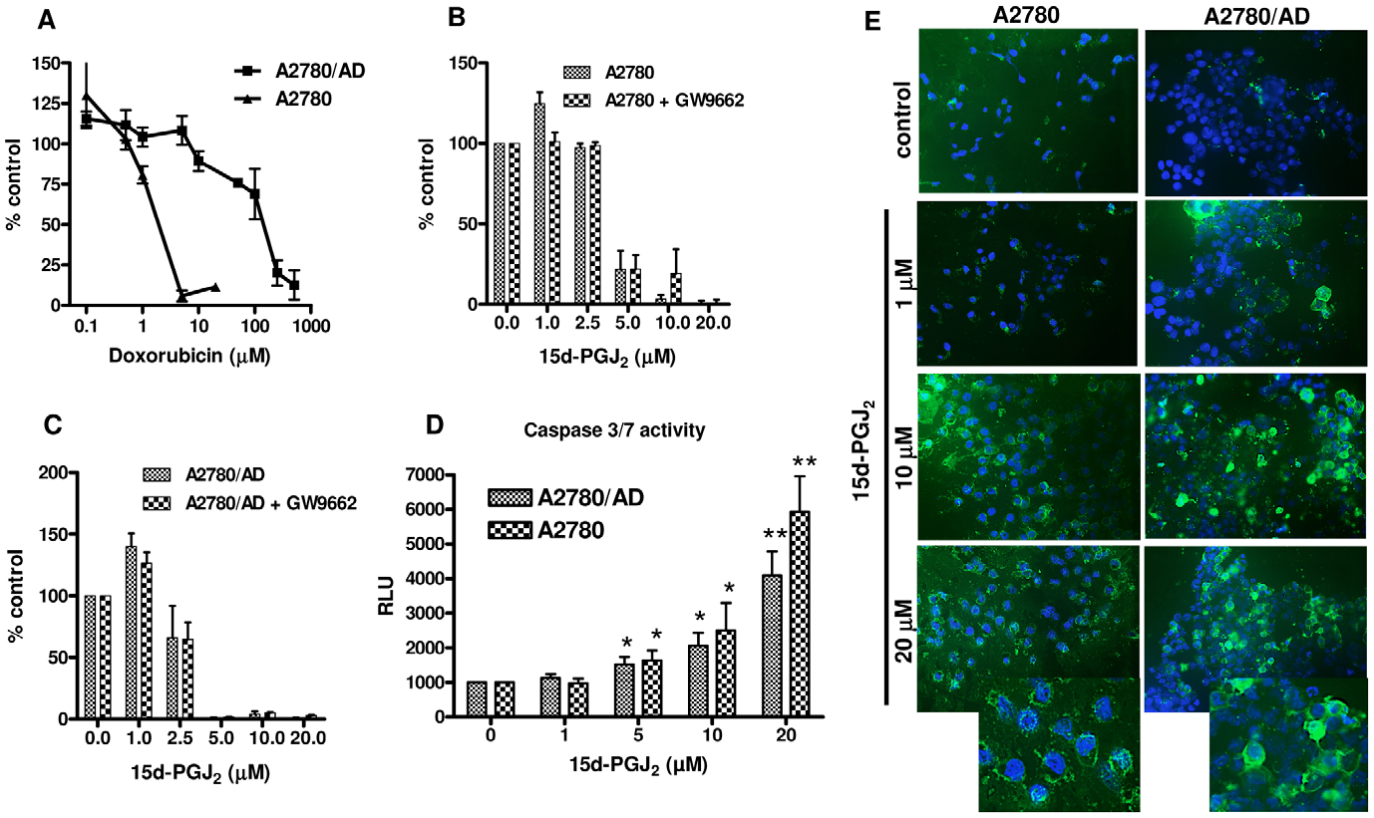
Effect of 15d-PGJ_2_ on the viability of A2780 and A2780/AD cells. (**A**) Doxorubicin killed A2780 cells completely at 5 µM whereas A2780/AD cells were highly resistant to doxorubicin. In contrast, 15d-PGJ_2_ induced cell death in both wild type A2780 (**B**) and doxorubicin-resistant A2780/AD (**C**) cell lines dose-dependently after 48 h. These effects were not PPARγ dependent as pre-incubation with an irreversible PPARγ antagonist GW9662 (10 µM) did not block these effects. Data are presented as % of control which was calculated by assuming untreated cells 100% viable as determined with the alamar blue assay. (**D**) 15d-PGJ_2_ enhanced caspase 3/7 enzymes activity (at t = 5.5 h) in both cell types in a concentration-dependent manner. Data represent mean ± SEM of the average of at least 3 separate experiments. Differences versus controls are shown as *p<0.05, **p<0.01. (**E**) Representative photomicrographs showing annexin V staining (green color), an indicator of apoptosis induction, in A2780 and A2780/AD cells lines after the treatment with 15d-PGJ_2_ concentration-dependently. DAPI staining (blue color) indicates the total number of cells. Magnification, 200×. The boxes show the images at higher magnification of 480×.

We confirmed that A2780/AD cells are highly resistant to doxorubicin as compared to wild-type A2780 cells as the IC_50_ of doxorubicin in A2780 and A2780/AD were 1.3 and 120 µM, respectively (Fig. 1A). However, treatment with 15d-PGJ_2_ caused cell death in both A2780 cells (IC_50_ = 4.3 µM) and A2780/AD cells (IC_50_ = 2.6 µM) after 48 h of incubation. 15d-PGJ_2_ is a known PPARγ agonist but we found that induction of cell death in both cell types was PPARγ-independent as pretreatment with the irreversible PPARγ antagonist GW9662 did not reverse the effects of 15d-PGJ_2_ neither in wild-type (Fig. 1B) nor in resistant (Fig. 1C) cell lines. Furthermore, we found that 15d-PGJ_2_ induced apoptosis in both wild-type and doxorubicin-resistant cells through activation of the caspase pathway as caspase-3/7 enzyme activities were significantly induced by 15d-PGJ_2_ in these cells after 5.5 h of incubation (Fig. 1D). Although A2780/AD (resistant cells) were slightly more sensitive to 15d-PGJ_2_ in the cell viability assay, there was slightly lower induction of caspase activity in these cells compared to A2780 cells, indicating participation of caspase-independent assays. The induction of apoptosis in these cells was also confirmed with annexin V staining, a marker for early apoptosis. We found that both cell types became positive for annexin V staining (green color) after the treatment with 15d-PGJ_2_ and the number of positive cells was increased at higher concentrations in both cell lines (Fig. 1E).

### 15d-PGJ_2_ induces apoptosis by inhibiting NF-κB pathway

**Figure 2:**
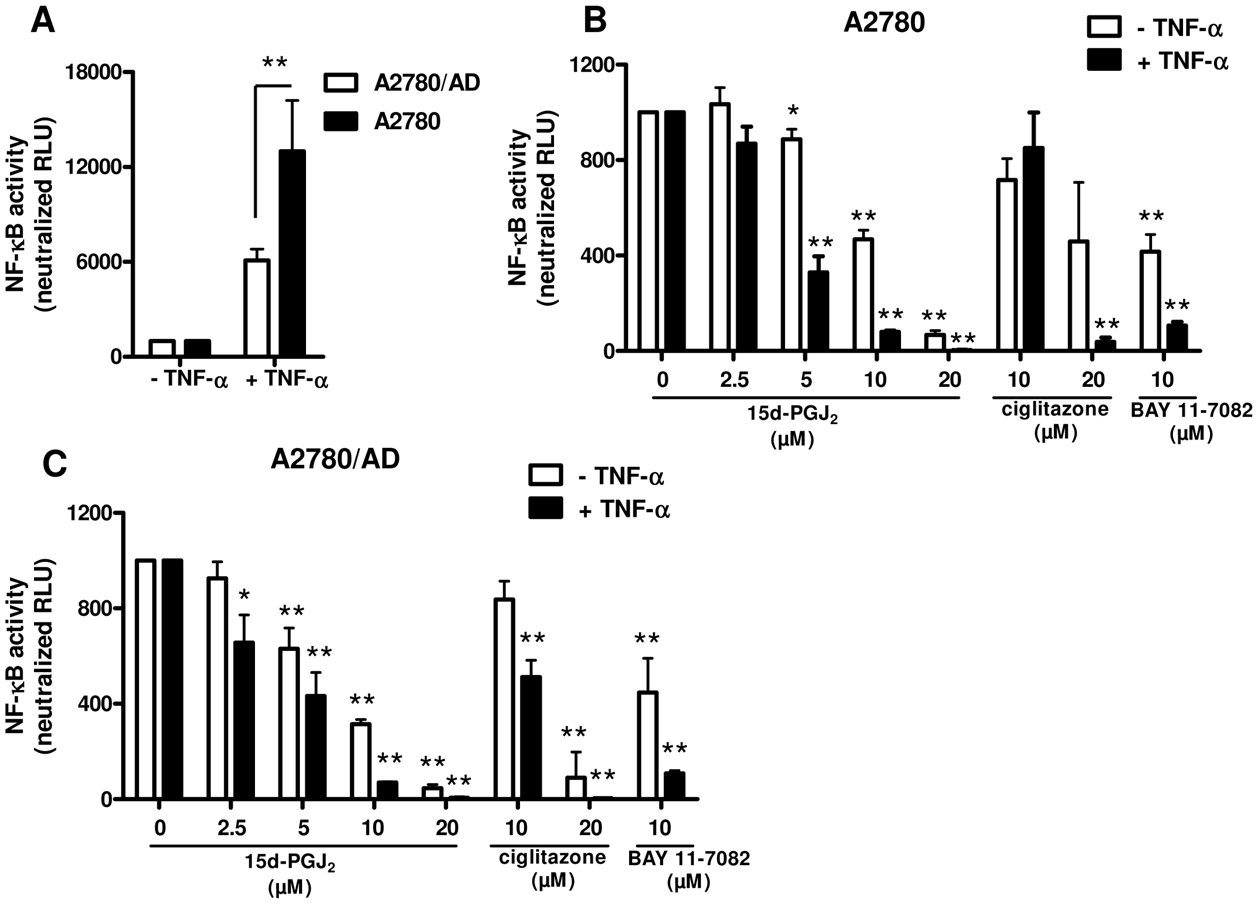
Inhibition of NF-κB activity by 15d-PGJ_2_ in A2780 and doxorubicin A2780/AD cells. To measure NF-κB activity, both A2780 and A2780/AD cells were transiently transfected with a plasmid containing the NF-κB promoter with a luciferase reporter element (pNF-κB-Luc) for 24 h. After 24 h, 15d-PGJ_2_, ciglitazone or BAY-11-7082 were incubated with and without TNF-α for 4 h and then luciferase activity was measured using a luminescence assay to determine the NF-κB activity. (**A**) The NF-κB pathway activator, TNF-α (100 ng/ml), induced the NF-κB activity in both cell types although the increase was higher in A2780 cells. Treatment with 15d-PGJ_2_ significantly inhibited NF-κB activity in both A2780 (**B**) and A2780/AD cells (**C**) which was more pronounced in TNF-α−activated cells. Ciglitazone inhibited NF-κB only at higher concentrations or in TNF-α−activated A2780/AD cells. BAY-11-7082 inhibited the NF-κB activity in both cell types with similar efficacy. Data represent the average of at least 3 separate experiments. Statistical differences versus the respective controls are shown as *p<0.05 and **p<0.01.

Since the apoptosis induced by 15d-PGJ_2_ was PPARγ-independent, we investigated the involvement of the NF-κB pathway, an important regulator of cell survival and proliferation, in A2780 and A2780/AD cells using the NF-κB luciferase reporter assay. We induced the NF-κB activity in these cells with human recombinant TNF-α which is a direct activator of the NF-κB pathway. We found that treatment with TNF-α (100 ng/ml) significantly enhanced the NF-κB activity in both cell types but more pronouncedly in A2780 (13-fold) compared to A2780/AD (6.0-fold) (Fig. 2A). Treatment with 15d-PGJ_2_ significantly reduced the basal and TNF-α-induced NF-κB activity in both cell types in a concentration-dependent manner (Fig 2B and 2C). Yet, the inhibitory effects were stronger in A2780/AD than A2780 as can be noticed at 2.5 and 5.0 µM concentrations. These data indicate that apoptosis-inducing effects of 15d-PGJ_2_ were mediated through NF-κB pathway. In addition, the PPARγ agonist ciglitazone did not inhibit the basal NF-κB activity at 10 µM concentration but only at higher concentrations. In further experiments with ciglitazone we therefore used concentrations that induce only PPARγ-specific effects (EC_50_ = 3.0 µM) [12] and not NF-κB-mediated effects. A well-known NF-κB-specific inhibitor, i.e. BAY-11-7082 inhibited the NF-κB activity in both cell types similarly and this inhibitor was used in further experiments to determine the effect of NF-κB inhibition in these cells.

### 15d-PGJ_2_ reduces the mRNA expression of Bcl-2, Bcl-xl, MDR1 and SIRT1

**Figure 3:**
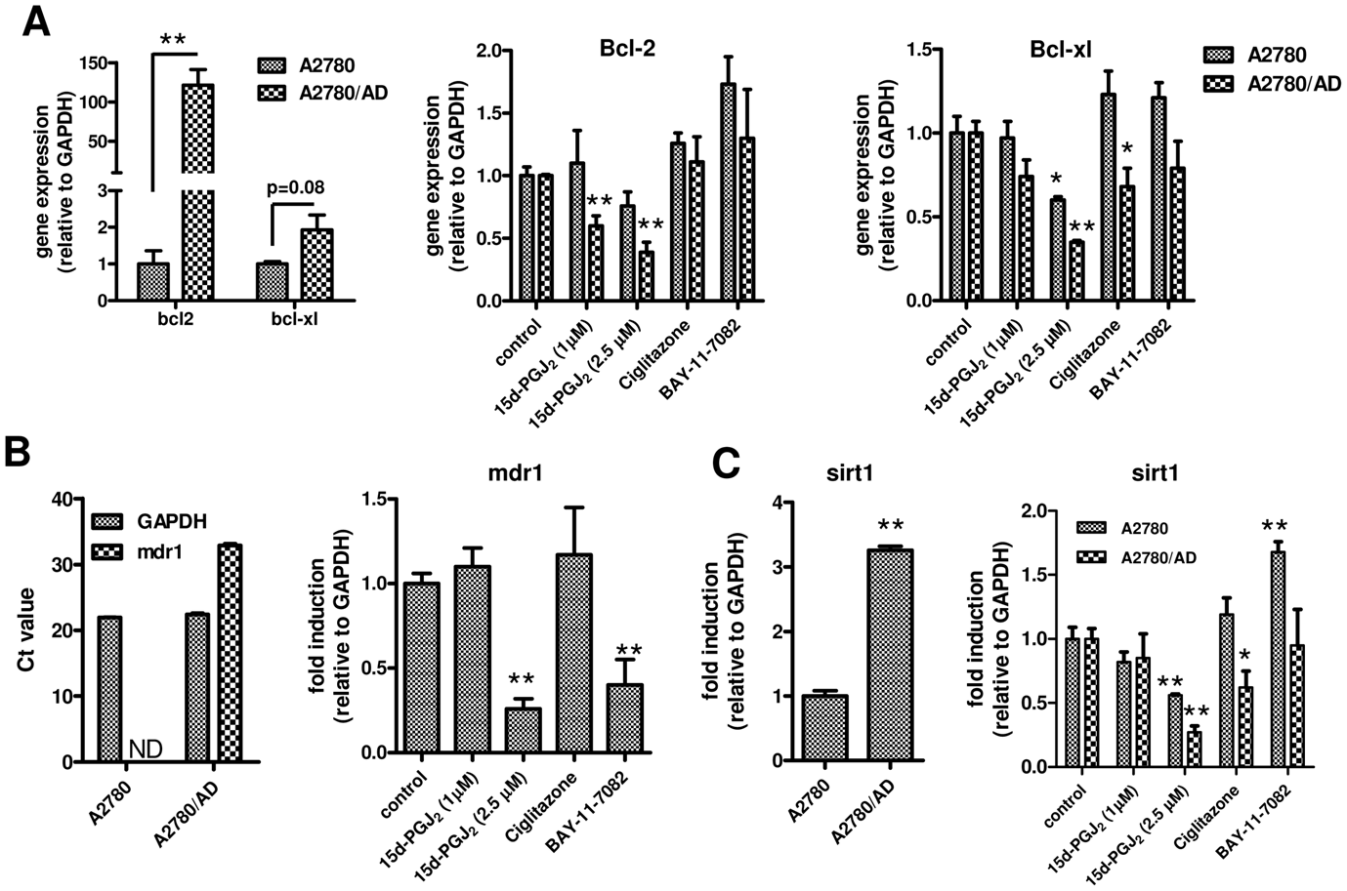
Effect of 15d-PGJ_2_ on the gene expression of Bcl-2, Bcl-xl, MDR1, and SIRT1. (**A**) A2780/AD cells had higher gene expression of Bcl-2 and Bcl-xl compared to A2780 cells. Effects of 15d-PGJ_2_, ciglitazone and BAY-11-7082 on the gene expression were determined after 48 h of incubation in both cell types. A2780/AD cells also had higher expression of MDR1 (**B**) and SIRT1 (**C**) compared to A2780 cells. Mdr1 gene expression was not detectable (ND) in A2780 cells. Effects of 15d-PGJ_2_, ciglitazone and BAY-11-7082 on MDR1 expression were determined only in A2780 cells (**B**) while the effects on SIRT1 expression were measured on both cell types (**C**). Data represent mean ± SEM for at least 3 experiments. Statistical differences versus the respective controls are shown as *p<0.05 and **p<0.01.

After gene expression analyses, we found that A2780/AD cells had substantial induction of anti-apoptotic genes such as Bcl-2 (120-fold) and Bcl-xl (2-fold) compared to non-resistant A2780 cells (Fig. 3A). Treatment with low concentrations of 15d-PGJ2 (1.0 and 2.5 µM) for 48 h reduced the Bcl-2 and Bcl-xl expression levels in A2780/AD more strongly than in A2780 cells (Fig. 3A), which might also explain the higher cell killing potency in A2780/AD cells. These effects were not due to PPARγ agonistic activity or NF-κB inhibitory effects of 15d-PGJ_2_ as the well-known PPARγ agonist ciglitazone and selective NF-κB inhibitor only had minor effect compared to 15d-PGJ_2_ (Fig. 3A).

In addition, we found that there was an up-regulation of multidrug resistance (MDR1) and class-III HDAC sirtuin-2 homologue (SIRT1) mRNA expression in doxorubicin-resistant A2780/AD cells compared to wild-type A2780 cells (Fig. 3B and 3C). The MDR1 levels in A2780 cells were below detection levels. Interestingly, treatment with 15d-PGJ_2_ significantly inhibited the MDR1 expression in the resistant cells and these effects were most likely attributed to its NF-κB inhibitory effects as BAY-11-7082 also inhibited the expression, whereas ciglitazone had no effect (Fig. 3B). 15d-PGJ_2_ also inhibited the SIRT1 expression in both cell types but these inhibitory effects were not seen with BAY-11-7082 (Fig. 3C). On the other hand, ciglitazone reduced the SIRT1 expression only in A2780/AD where SIRT1 levels were elevated.

### 15d-PGJ_2_ inhibits wound healing process

**Figure 4:**
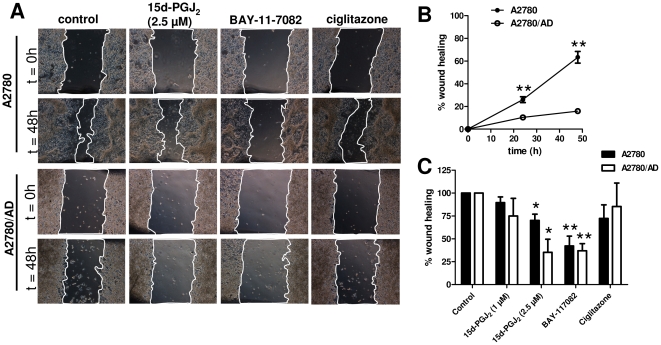
Effect of 15d-PGJ_2_ on wound healing process. To determine the effects of 15d-PGJ_2_ on cell migration, a wound healing assay was performed on A2780 and A2780/AD cells as described in materials and methods. (**A**) Representative pictures showing the scratch (wound) at t = 0 h and t = 48 h with/without different treatments. A mark (visualized in these pictures on the upper or bottom side) was placed to locate the same area on the scratch, pictures were made using an inverted microscope and the open area was calculated at the indicated time points. Magnification, 40×. (**B**) The graph shows % wound healing in A2780 and A2780/AD cells without treatments. A2780 cells had higher migration rate compared to A2780/AD cells. Data represent mean ± SEM for 3 different experiments. **p<0.01 versus A2780/AD. (**C**) Treatment with 15d-PGJ_2_ and BAY-11-7082 inhibited the wound healing response after 48 h incubations while ciglitazone did not show any inhibitory effects. Data represent mean ± SEM for 3 different experiments. *p<0.05 and **p<0.01 versus values at t = 0 h.

Since the chemotherapeutic resistant cells may have different migration behavior than its wild-type version, we furthermore examined the effect of induced resistance on these parameters using *in vitro* wound healing assay. The assay typically measures migration of cells by measuring the closure of a standard scratch in time. We found that A2780/AD cells had significantly slower migration than A2780 cells (Fig. 4A and 4B). 15d-PGJ_2_ reduced the migration of both cell types in a dose-dependent manner (Fig. 4C). Although 15d-PGJ_2_ reduced the cell viability in A2780/AD cells at 2.5 µM concentration (Fig. 1C), we did not see cell death in these experiments which might be due to confluent cell layer used in these experiments. We found earlier that in confluent cell layer 15d-PGJ_2_ had no effect on the cell viability of A2780/AD (data not shown). Absence of cell death in these experiments is also apparent in the represented pictures (Fig. 4A). The inhibitory effects on wound healing were also seen with BAY-11-7082 but not with ciglitazone indicating the effects of 15d-PGJ2 might be due to its NF-κB inhibitory effects.

### Effect of 15d-PGJ_2_ on cell morphology

**Figure 5:**
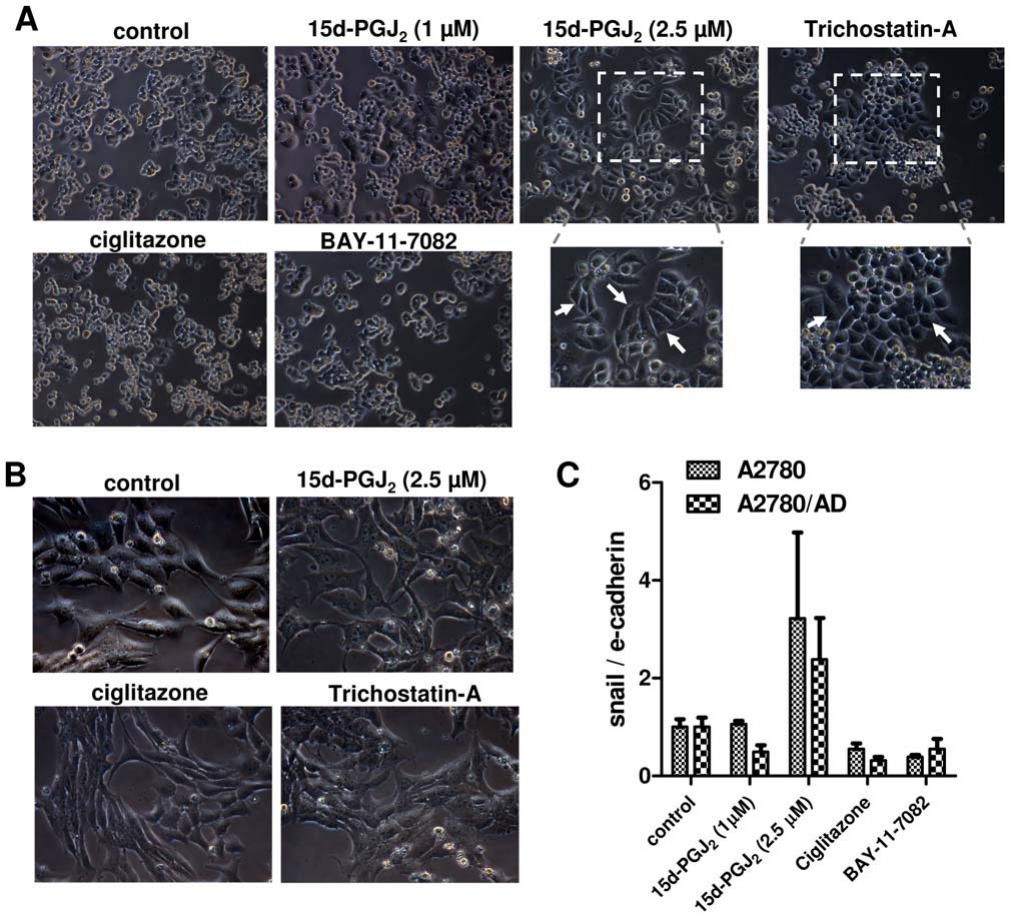
Effect of 15d-PGJ_2_ on cell morphology. (**A**) Representative pictures showing the change in cell morphology of A2780/AD after incubation with 15d-PGJ_2_ (2.5 µM) and trichostatin A (70 nM). In contrast, other treatments such as ciglitazone and BAY-11-7082 did not affect the morphology. Arrows point out the transformed cells that had more cytoplasm and epithelial cells structure. Magnification, 200× (**B**) Representative pictures of A2780 cells after incubation with different treatments. Treatment with 15d-PGJ_2_ (2.5 µM) and trichostatin A (70 nM) led to a clear increase in the cytoplasm. Magnification, 200× (C) Ratio of snail to e-cadherin gene expression in A2780 and A2780/AD cells. Data represent mean ± SEM for 3 experiments.

During the incubations with 15d-PGJ_2_, we noticed that cells were transformed into enlarged and elongated cells. This phenotypic change was most prominently visible in the dox-resistant A2780/AD cells. These cells were initially rounded and shrunk but after transformation they attained more cytoplasm with distinct cellular boundaries and displayed an appearance of epithelial-like structure (Fig. 5A). Treatment with ciglitazone or BAY-11-7082 induced no change in cell morphology indicating no role of PPARγ and NF-κB pathways. Since it was reported in the literature that the HDAC inhibitor trichostatin A could transform the ovarian carcinoma cells [13], we treated cells with trichostatin A and found that cells got transformed in a similar way as with 15dPGJ_2_ (Fig. 5A). Although A2780 cells also looked more stretched with 15d-PGJ_2_ and trichostatin A, differences versus control cells were not large (Fig. 5B).

Since the transformation of cells might be related to epithelial-mesenchymal transition (EMT) process, we examined the expression of snail and e-cadherin transcripts and found that the ratio of snail to e-cadherin was increased in both cell types after the exposure of 2.5 µM 15d-PGJ_2_ though the differences were not statistically significant. These data indicate that change in cell morphology induced by 15d-PGJ_2_ might be due to induction of the EMT process.

### 15d-PGJ_2_ inhibits HDAC 1, 2 and 3 mRNA expression

**Figure 6:**
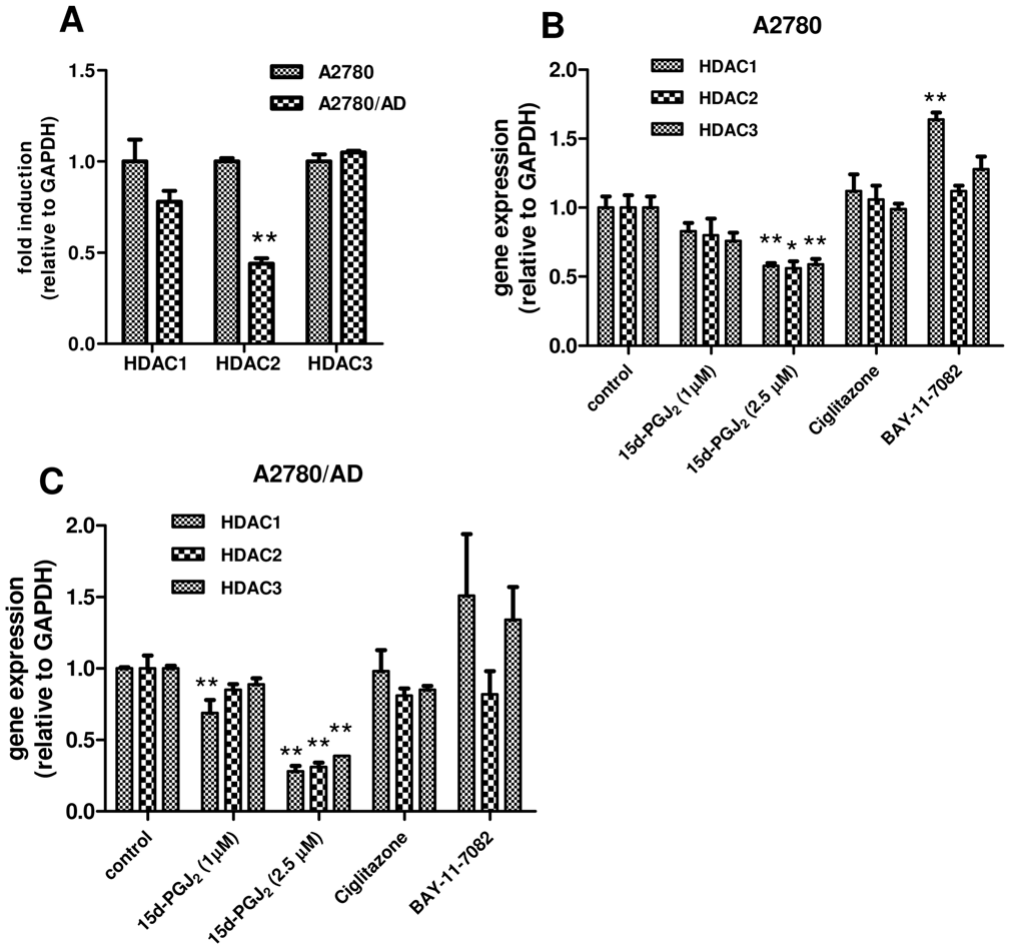
Effect of 15d-PGJ_2_ on the gene expression of HDAC1, 2, and 3. (**A**) Real-time qPCR data show the differences between the basal gene expression levels of HDAC 1, 2, and 3 in A2780 and A2780/AD cells. **p<0.05 versus A2780 cells. Effect of different treatments on HDAC genes were determined in A2780 (**B**) and A2780/AD cells (**C**) after 48 h incubation. Data represent mean ± SEM for 3 separate experiments. Statistical differences versus the respective controls are shown as *p<0.05 and **p<0.01.

Since the cell transformation effects of 15d-PGJ_2_ were also induced by the HDAC inhibitor, we examined the effect of 15d-PGJ_2_ on HDAC in both cell types. First, we compared the mRNA levels of HDAC1, 2 and 3 in A2780 and A2780/AD cells and found that HDAC2 was significantly downregulated in the resistant cells compared to wild-type cells (Fig. 6A). Treatment with 15d-PGJ_2_ inhibited the HDAC1, 2 and 3 mRNA expressions in both cell types dose-dependently (Fig. 6B and 6C). The effects were most pronounced in resistant cells. These inhibitions were not found with ciglitazone and BAY-11-7082, yet there was an increase in HDAC1 expression after NF-κB inhibition with BAY-117082.

### 15d-PGJ_2_ inhibits SIRT1 and HDAC enzymes activities due to its electrophilic carbon atoms

**Figure 7:**
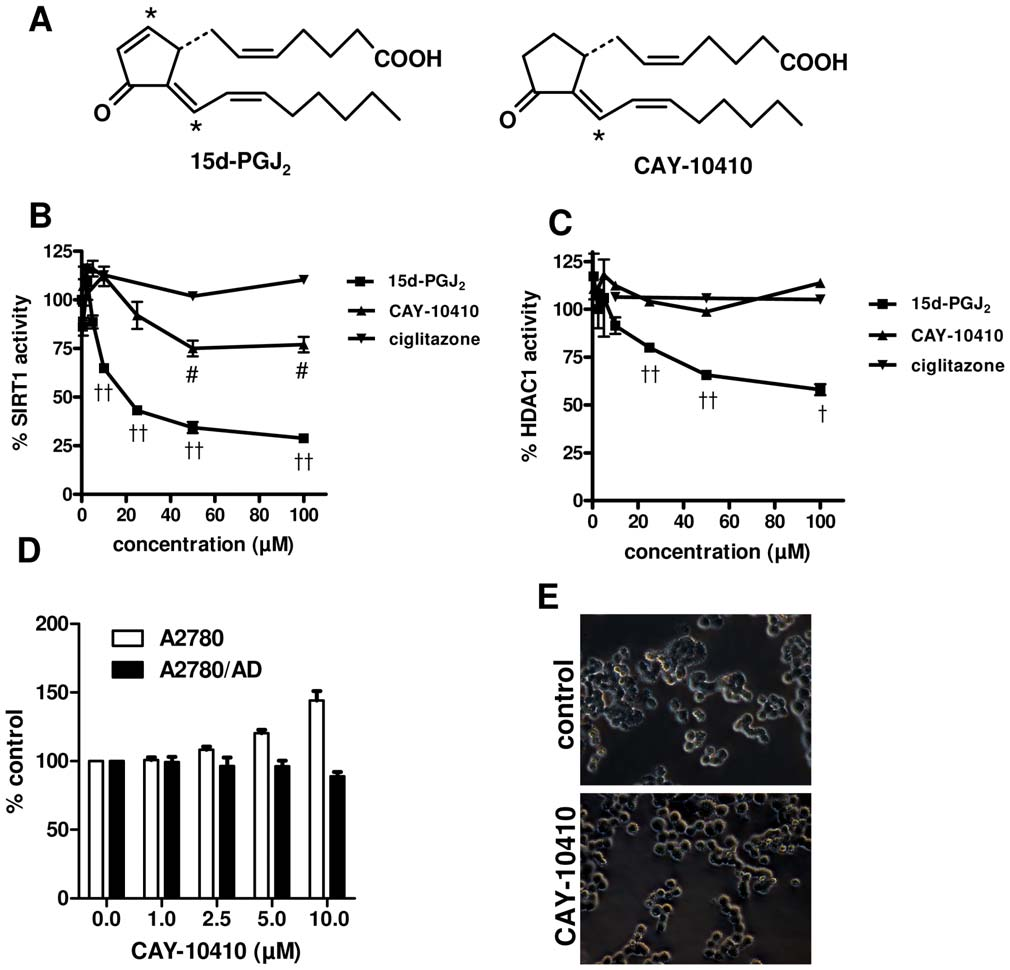
Effect of 15d-PGJ_2_ and its analog CAY-10410 on SIRT1 and HDAC1 activity. (**A**) Chemical structures of 15d-PGJ_2_ (15-deoxy-Δ^12,14^-Prostaglandin J_2_) and its analog CAY-10410 (9,10-dihydro-15-deoxy-Δ^12,14^-Prostaglandin J_2_). Asterisk indicates the electrophilic carbon atoms which might be involved in Michael addition reaction. Panel (**A**) and (**B**) show the concentration-dependent effects of 15d-PGJ_2_, CAY-10410 and ciglitazone on the deacetylase activity of SIRT1 and HDAC1, respectively using *in vitro* enzyme assays. ^#^P<0.01 versus ciglitazone, ^†^p<0.05 and ^††^p<0.01 versus CAY-10410. (**D**) Effect of CAY-10410 on the cell viability of A2780 and A2780/AD cells as measured with an alamar blue assay. (**E**) Treatment with CAY-10410 (2.5 µM) did not induce cell transformation after 48 h incubation. Magnification, 200×.

Since we found that 15d-PGJ_2_ could inhibit the gene expression of SIRT1 and other HDACs, we wondered whether 15d-PGJ_2_ was able to inhibit these enzyme activities directly. We used a p53 sequence-based substrate (Arg-His-Lys-Lys(e-acetyl)-AMC) assay to determine SIRT1 activity and for HDAC1 we used an acetylated lysine substrate-based assay. In order to find whether the C9 electrophilic carbon atom is responsible for the activity, we included the 15d-PGJ_2_ analog CAY-10410 that lacks the electrophilicity at C9 (Fig. 7A). We found that 15d-PGJ_2_ inhibited the SIRT1 activity dose-dependently with up to 71% inhibition whereas CAY-10410 only showed only moderate inhibition of 23% while ciglitazone showed no inhibition at all (Fig. 7B). In the HDAC1 enzyme assay, 15d-PGJ_2_ led to 40% inhibition but CAY-10410 and ciglitazone did not show any inhibition (Fig. 7C).

We further examined whether the effects of 15d-PGJ_2_ were displayed due to the C9 electrophilic atom, we tested the effects of CAY-10410, lacking electrophilicity at C9 carbon atom, on cell viability and transformation. We found that CAY-10410 did neither induce any cell death nor transformation of the cells (Fig. 7D and 7E).

## Discussion

In the present study, we have demonstrated the effects of 15d-PGJ_2_, a product of cyclo-oxygenase activity and as an endogenous PPARγ agonist, on apoptosis, drug resistance, cell migration and transformation of cancer cells. We examined its effects on wild-type as well as doxorubicin-resistant ovarian cancer cells. We hypothesized that if 15d-PGJ_2_ affects multi-drug resistant tumor cells, it is important to explore its mechanism of action because it may lead to new targets for intervention in therapy-resistant cells. In literature, multiple mechanisms of action of 15d-PGJ_2_ have been shown which are related to either PPARγ−dependent pathways or PPARγ−independent pathways such as NF-κB, p53, and Nrf2/keap1 [11]. In the present study, we have profound effects of this cyclo-oxygenase product on tumor cells and we showed that these different actions are mediated by different mechanisms of action of 15d-PGJ_2_. Most importantly, we discovered the new mechanisms of action of 15d-PGJ_2_ i.e. inhibition of SIRT1 and other histone deacetylases (HDAC) which regulate multiple mechanisms in cancer cells. We furthermore elucidated that 15d-PGJ_2_ inhibits the activity of these enzymes due to its electrophilic C9 carbon atom.

Development of resistance against chemotherapy is a big challenge in medicine and therefore new therapeutics are a prerequisite for resistant cancers [14]. The apoptosis-inducing effects of 15d-PGJ_2_ through PPARγ-independent pathways, in non-resistant tumor cells, have been well reported in many studies using different assays such as caspase, annexin V staining and mitochondrial activity assays [4], [6], [15]. However, in this study we show that 15d-PGJ_2_ can also induce apoptosis in doxorubicin−resistant A2780/AD cells through PPARγ-independent pathways. Tumor cells can induce drug resistance through inducing drug efflux pump (p-glycoprotein or MDR1), by up-regulation of anti-apoptotic genes (Bcl-2, Bcl-xl) or by other mechanisms [16]. Indeed, we found up-regulation of MDR1, Bcl-2 and Bcl-xl expression in doxorubicin-resistant A2780/AD cells compared to the non-resistant cells (Figure 3A, 3B). Since 15d-PGJ_2_ induced apoptosis in these A2780/AD cells, it is apparent that up-regulation of these pathways does not affect the activity of 15d-PGJ_2_. On the contrary, 15d-PGJ_2_ inhibited the gene expression of MDR1, Bcl-2 and Bcl-xl in the resistant cells. These data suggest that 15d-PGJ_2_ is in potential to kill a variety of chemotherapy resistant tumor cells.

NF-κB is a ubiquitous transcription factor that regulates cell proliferation and cell survival by controlling the caspase family of enzymes [9]. 15d-PGJ_2_ directly inhibits the NF-κB activity by inhibiting the degradation of IκB that prevents translocation of NF-κB units into the nucleus [8]. In our previous study, we have shown that 15d-PGJ_2_ inhibited NF-κB in different tumor cells [4] and in this study we therefore compared the effect of 15d-PGJ_2_ on this pathway in doxorubicin-resistant and non-resistant tumor cells. Although 15d-PGJ_2_ inhibited NF-κB activity in both A2780 and A2780/AD cells, the effects were significantly more pronounced when this pathway was activated by TNF-α. Using a well-known PPARγ agonist, ciglitazone, or the irreversible NF-κB−specific inhibitor, BAY-11-7082, we examined whether the effects of 15d-PGJ_2_ were mediated through PPARγ or NF-κB. Our data demonstrate that inhibition of MDR1 gene expression and cell migration in wound healing assay by 15d-PGJ_2_ were most likely mediated through the NF-κB pathway as inhibition of this pathway using BAY-11-7082 resulted in similar effects. Also other studies found that NF-κB can induce MDR1 expression [17] and cell migration and metastasis in tumor cells [18]. In contrast, no effects of the PPARγ agonist ciglitazone were found. Apparently, there was almost no participation of PPARγ in these effects. These data indicate that 15d-PGJ2 may diminish drug resistance and inhibit tumor metastasis due to its NF-κB inhibitory action. These effects were already seen at low concentrations, indicating that this activity of 15d-PGJ2 might be physiologically relevant.

SIRT1 is a NAD^+^-dependent deacetylase which deacetylates histones and nonhistone proteins and has been shown to delay senescence [19]. Chu et al have shown that ectopic over-expression of SIRT1 induces the expression of MDR1 and leads to resistance to chemotherapy in tumor cells [10]. In line with these data, we also found an enhanced SIRT1 expression in the resistant cells. This increase in SIRT1 was significantly reduced by 15d-PGJ_2_. This inhibitory effect might be due to the PPARγ agonistic activity as the PPARγ agonist, ciglitazone, also inhibited the expression of SIRT1 in the resistant cells. In accordance with this, a recent study has demonstrated that PPARγ and SIRT1 mutually regulate each other and PPARγ inhibits the SIRT1 activity [20]. Inhibition of SIRT1 by 15d-PGJ_2_ was not due to its NF-κB inhibiting activity as BAY-11-7082 did not inhibit the SIRT1 expression. In contrast, it led to an increase in SIRT1 expression in A2780 cells. This increase might be due to a feedback loop between SIRT1 and NF-κB as over-expression of SIRT1 is known to inhibit NF-κB by deacetylating RelA/p65 unit [21]. In addition, next to its effect on gene expression, 15d-PGJ_2_ also strongly inhibited the SIRT1 enzyme activity in vitro (Figure 7B) supporting its direct effects on SIRT1-mediated pathways. This is the first study showing the effects of this cyclo-oxygenase product on SIRT1 and since SIRT1 blocks senescence, cell differentiation and stress-induced apoptosis and promotes cell growth and drug resistance [19], the effects of 15d-PGJ_2_ shown in this study on SIRT1 are physiologically very relevant.

We observed a change in cell morphology after treatment with 15d-PGJ_2_, which led us to the hypothesis that 15d-PGJ_2_ was not only inhibiting class III HDAC (SIRT1) activity but also class I HDAC enzymes. It was known that an inhibitor of class I HDAC activity, trichostatin A, can induce transformations in cell morphology [13] which were quite similar to the changes seen in the present study with 15d-PGJ_2_ (Fig. 5). This was confirmed when 15d-PGJ_2_ was found to inhibit HDAC 1, 2 and 3 gene expression levels in both cell types and also reduced the deacetylase activity of HDAC1 *in vitro*. The HDAC inhibition was exclusive for 15d-PGJ_2_ as ciglitazone and BAY-11-7082 did not inhibit the expression of these genes. Since the potency of 15d-PGJ_2_ on SIRT1 gene expression and activity was higher than its potency on HDAC1 gene expression and activity, the pharmacological effects of 15d-PGJ_2_ at lower doses may be predominated by its SIRT1-inhibitory actions. Moreover, the morphological changes might be related to the induction of EMT process as the snail to e-cadherin ratio was induced with 15d-PGJ_2_. Snail is the transcription factor for suppressing e-cadherin expression, and the higher ratio between them has been described earlier indicating the overall lower survival of patients with ovarian and breast carcinoma [22], [23].

Many studies have shown that 15d-PGJ_2_ exhibits most of its pharmacological effects by conjugating to a nucleophile moiety in a protein such as a free cysteine group, through a Michael addition reaction [11]. In this study, we demonstrated that electrophilic carbon atoms of 15d-PGJ_2_ were also responsible for the inhibitory effects on SIRT1 and HDAC1 enzymes. Using an analogue of 15d-PGJ_2_,, CAY-10410, that lacks electrophilicity at the C9 atom, we revealed that HDAC inhibition was completely dependent on the C9 atom while SIRT1 inhibition was mostly dependent on C9 but also slightly on the C13 atom as CAY-10410, having electrophilicity at C13, also showed some degree of SIRT1 inhibition. Lack of activity of CAY-10410 in cell viability assays and cell transformation indicates that electrophilicity of C9 atom is highly important for the major activities of 15d-PGJ_2_.

In summary, 15d-PGJ_2_ is a strong apoptotic−inducing arachidonic metabolite which induced cell death in doxorubicin−resistant human ovarian carcinoma cells similar to non-resistant cells. Many effects of 15d-PGJ_2_ were induced through its NF-κB inhibiting activity and were not due to its PPARγ-agonistic activity. In addition to this, we found direct effects on SIRT1 and HDAC1 expression and activity, mediated by its C9 atom, which represent new mechanisms of action of this endogenous mediator. These intracellular effects affected tumor cell migration, transformation, and attenuated MDR1 gene expression and thus therapy resistance. The present study will help better understand the complexity of actions of this endogenous metabolite. Moreover, the knowledge on its inhibitory activity on SIRT1 and HDAC may help in designing their new inhibitors. As showed in our recent study that pharmacokinetics of 15d-PGJ_2_ is controlled by its albumin binding and tumor vasculature [4], in order to apply 15d-PGJ_2_ for therapeutic purpose it would be highly interesting to employ a tumor drug delivery approach to improve its pharmacokinetics and to achieve tumor-specific effects.
